# Sensitivity and Specificity of Selected Biomarkers and Their Combinations in the Diagnosis of Ovarian Cancer

**DOI:** 10.3390/diagnostics14090949

**Published:** 2024-04-30

**Authors:** Aleksandra Englisz, Marta Smycz-Kubańska, Aleksandra Mielczarek-Palacz

**Affiliations:** 1 The Doctoral School, Medical University of Silesia, 40-055 Katowice, Poland; d201166@365.sum.edu.pl; 2 Department of Immunology and Serology, Faculty of Pharmaceutical Sciences in Sosnowiec, Medical University of Silesia, 40-055 Katowice, Poland; mkubanska@sum.edu.pl

**Keywords:** ovarian cancer, biomarkers, sensitivity, specificity

## Abstract

One of the greatest challenges in modern gynecological oncology is ovarian cancer. Despite the numerous studies currently being conducted, it is still sometimes detected at late clinical stages, where the prognosis is unfavorable. One significant contributing factor is the absence of sensitive and specific parameters that could aid in early diagnosis. An ideal screening test, in view of the low incidence of ovarian cancer, should have a sensitivity of greater than 75% and a specificity of at least 99.6%. To enhance sensitivity and specificity, diagnostic panels are being created by combining individual markers. The drive to develop better screening tests for ovarian cancer focuses on modern diagnostic methods based on molecular testing, which in turn aims to find increasingly effective biomarkers. Currently, researchers’ efforts are focused on the search for a complementary parameter to those most commonly used that would satisfactorily enhance the sensitivity and specificity of assays. Several biomarkers, including microRNA molecules, autoantibodies, cDNA, adipocytokines, and galectins, are currently being investigated by researchers. This article reviews recent studies comparing the sensitivity and specificity of selected parameters used alone and in combination to increase detection of ovarian cancer at an early stage.

## 1. Introduction

The 2020 GLOBOCAN study documented 313,959 new occurrences of ovarian cancer and 207,252 deaths from the disease. This puts it as the seventh most prevalent kind of cancer globally and the fifth leading cause of cancer deaths worldwide [1]. Ovarian cancer (OC) carries a high mortality rate as a result of symptoms that appear late and lack specificity, including bloating, gas pain, back pain, indigestion, and early satiety. They are frequently misinterpreted as symptoms of other diseases, which can considerably postpone the initiation of treatment [2]. The survival rate of women relies on the stage of disease diagnosed. Stage I has a five-year survival rate of 93%, followed by 68% for stage II, 27% for stage III, and 13.4% for stage IV [3]. The lack of sensitive and effective markers for early detection of ovarian cancer leads to high mortality rates. The diagnostic utility of a screening test is reliant on its positive predictive value (PPV), which refers to the proportion of women with a positive result on the screening test who actually have ovarian cancer. The PPV is influenced by sensitivity (SN), i.e., the percentage of women with ovarian cancer who test positive, specificity (SP), i.e., the percentage of women without ovarian cancer who test negative, and the prevalence of ovarian cancer in the study population [4]. The ideal screening marker for cancer must have sufficient specificity and sensitivity to attain a positive predictive value (PPV) of 10%, indicating that 1 diagnosed cancer will be found for every 10 positive test outcomes [5]. To ensure an effective screening test for ovarian cancer, a sensitivity greater than 75% and a minimum specificity of 99.6% are required [5,6].

Using the medical database PubMed, we performed a literature search using key words such as ovarian cancer, biomarkers, CA125, sensitivity, and specificity. The aim of this study was to review recent studies comparing the sensitivity, specificity, or AUC of selected parameters used alone and in combination to improve the detection of early-stage ovarian cancer. The parameters analyzed included those already used in the diagnosis of ovarian cancer, such as CA125 or HE4, as well as biomarkers of emerging interest to researchers. A literature review was conducted comparing the sensitivity, specificity, or AUC of a single CA125 assay and its combination with selected markers to identify the combination with the highest values.

## 2. Diagnostic Utility of the CA125 Test

The CA125 antigen is one of the most extensively researched markers employed in ovarian cancer diagnosis. However, its use in clinical practice faces controversies resulting from the sensitivity and specificity of the test [7]. While its applicability in early-stage screening for ovarian cancer is uncertain, it is utilized as an indicator to appraise chemotherapy effectiveness and assess prognosis [8,9]. Gupta D et al. [9], after reviewing the epidemiological literature, concluded that serum CA125 levels in serum are a powerful predictor of overall and progression-free survival in patients with OC. The researchers’ analysis of the data showed that decreasing levels of CA125 indicate a positive response to cancer therapy, while rising levels indicate a relapse of the cancer [9].

According to Zhang M. et al. [10], their research revealed a strong correlation between serum CA125 levels and tumor grade (*p* < 0.001). Low CA125 levels are linked to earlier cancer stages and a more favorable prognosis for patients [10]. Cramer D.W et al. [11] preliminarily found that the combination of CA125 with serum antibodies and the creation of circulating immune complexes (CICs) could cause low serum CA125 levels, potentially interfering with the results of serum CA125 testing [11]. Elevated CA125 levels may be present in various conditions, including but not limited to endometrial, breast, pancreatic, gastrointestinal, and lung cancers [12], as well as liver cirrhosis, pelvic inflammatory disease, and uterine fibroids [13]. About 1% of the healthy population and 5% of patients with menstrual or benign conditions such as endometriosis and coronary artery disease also have increased serum CA125 levels [10]. Elevated CA125 levels found in other physiological or pathological conditions result in a low specificity of the test (73–77%) [14]. The presence of certain chronic conditions, such as hypercholesterolemia, osteoporosis, and osteoarthritis, is linked to CA125 and may impact the analysis of CA125 findings in ovarian cancer screening [15]. Early-stage ovarian cancer shows heightened CA125 levels in just 50% of cases, while advanced disease displays elevated levels in 92% of cases [5]. Studies indicate that CA125 has a sensitivity of only 50–62% when diagnosing ovarian cancer in its early clinical stages [14]. A test developed in 1981 by Bast et al. [16] to define serum levels of CA125 showed elevated serum CA125 levels in 1% of healthy patients, 6% of patients with benign lesions, and 82% of patients with surgically confirmed OC.

### Cut-Off Point of the CA125 Test

A serum CA125 concentration above 35 U/mL was used as a cut-off point [16]. As per Sopik et al. [4], about 5% of healthy women exhibited CA125 levels of 35 U/mL or higher, 1% of healthy women displayed CA125 levels of 70 U/mL or higher, and 0.1% of healthy women illustrated CA125 levels of 100 U/mL or higher [4]. In contrast, CA125 levels did not rise above 35 U/mL in around 20% of EOC patients [10]. Wang Q et al. [17] demonstrated in a meta-analysis that elevated pretreatment CA-125 levels were significantly associated with worse overall and progression-free survival in patients with EOC [17].

**Figure 1:**
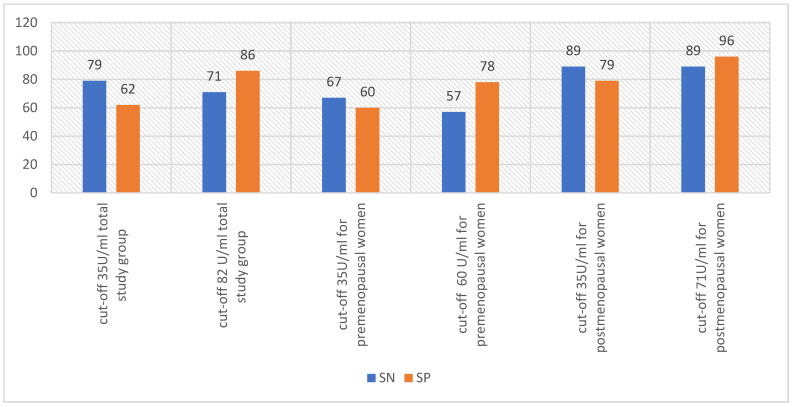
Sensitivity and specificity of CA125 when changing the standard cut-off of 35 U/mL to the cutoff values adopted by the investigators [18]; SN—sensitivity, SP—specificity.

**Figure 2:**
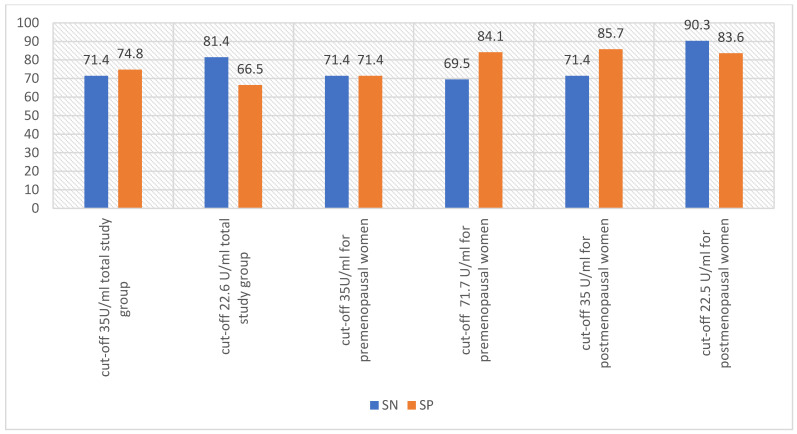
Sensitivity and specificity of CA125 when changing the standard cut-off of 35 U/mL to the cutoff values adopted by the investigators [19]; SN—sensitivity, SP—specificity.

One way to enhance the positive predictive value (PPV) of screening tests is by raising the test’s cut-off point, leading to decreased false positives [5]. According to Charkhchi et al. [5], it may be possible to diagnose early stage type II ovarian cancer at the point-of-care in primary care by using CA125 tests with a cut-off point higher than 35 U/mL [5]. Al Musalhi K et al. [18] found that raising the CA125 cut-off level significantly improved specificity but lowered sensitivity [18]. In a study by Kim B. et al. [19], the use of optimal limits instead of the standard limit (>35 U/mL) resulted in increased sensitivity of CA125 in the postmenopausal and general groups, but decreased specificity. However, in the premenopausal group, the sensitivity remained the same, while the specificity increased [19]. Figure 1 and Figure 2 show the changes in sensitivity and specificity of CA125 with changes in the test cut-off.

Sopik et al. [4] demonstrated that when the target group were limited to stage IIIa/IIIb and the test cut-off point was increased from 35 U/mL to 70 U/mL, the sensitivity of the test decreased from 75% to 70% for all cases of ovarian cancer, while the test specificity for healthy women increased to 99% [4].

Modified cut-off values for CA125 were useful in both premenopausal (CA125 = 61.60 U/mL) and postmenopausal women (CA125 = 6.21 U/mL) to exclude ovarian malignancy [20].

Changing the cut-off point of the CA125 test changes the sensitivity and/or specificity of the test. The search for optimal values, the menopausal status of patients should be considered. Finding optimal cut-off values for the CA125 test in the early stages of ovarian cancer is likely to improve the diagnosis and detection of this disease.

## 3. CA125 and Its Combinations with Other Parameters; Algorithms ROMA, ROMI, RMI and OVA1

To enhance the screening accuracy of the CA125 test, it is frequently paired with other assays. Alongside CA125, the most commonly utilized parameter for diagnosing ovarian cancer is HE4. As per an umbrella review conducted by Sun ML et al. [21], HE4 is the preferred biomarker for cancer prognosis. Additionally, clinicians can utilize HE4 as a biomarker to help diagnose and predict EC, OC, and LC (lung cancer) [21]. A meta-analysis carried out by Li J et al. [22] indicated that when histologic type and stage are not considered, the serum HE4 assay is a superior diagnostic tool for EC because of its increased sensitivity compared to the CA125 assay [22]. Similar findings were presented by Han Li-Na et al. [23], who conducted a meta-analysis assessing the link between HE4 and prognosis in EC. Their conclusion was that the HE4 marker may be a better predictor of EC than CA125, and high serum levels of HE4 were associated with poor OS and disease-free survival [23].

**Figure 3:**
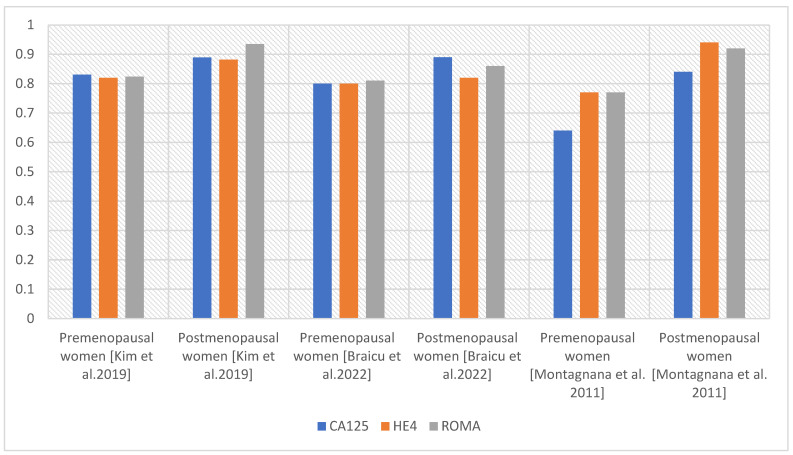
Comparison of AUC values for the parameters CA125, HE4, and ROMA in pre- and postmenopausal women [19,27,30].

**Figure 4:**
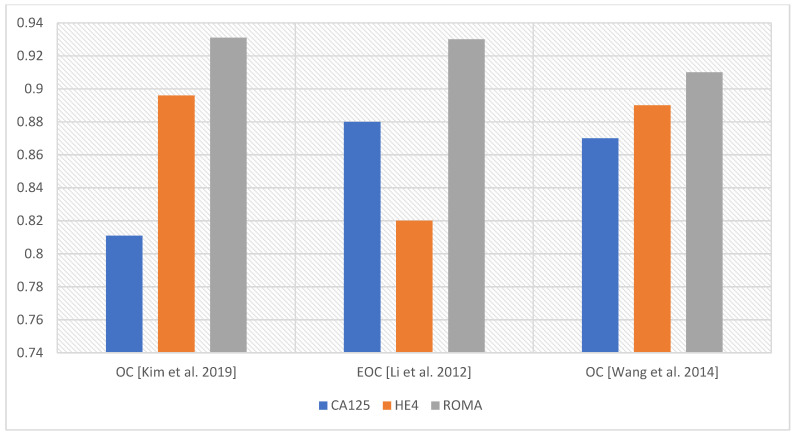
Comparison of AUCs for parameters CA125, HE4, and ROMA [19,28,29].

Algorithms, such as ROMA, ROMI, RMI, and OVA1, combining the determination of CA125 with other parameters, have been utilized for diagnosing ovarian cancer. The ROMA, risk of ovarian malignancy algorithm, was recognized by the Food and Drug Administration (FDA) in 2011 as a tool for assessment of risk of ovarian malignancy. It is composed of the determination of CA125 and HE4 levels and statistical estimation of malignancy risk, considering the patient’s menopausal status [24]. Janas Ł. et al. [25] confirmed the usefulness of CA125, HE4, and the ROMA algorithm for the pre-operative diagnosis of ovarian cancer. They emphasized HE4’s role in increasing diagnostic efficiency and verifying false-positive CA125 results, particularly in cases of endometriosis. In their study, they achieved a sensitivity and specificity of 93.2% and 71.5% for CA125, 95.4% and 81.3% for HE4, and 95.4% and 79.8% for the ROMA algorithm. In premenopausal women, CA125, HE4, and ROMA achieved sensitivities of 100% with specificities of 65.6%, 93.4%, and 82.0%, respectively. In the group of postmenopausal women, SN and SP reached values of 92.1% and 81.7% for CA125, 94.7% and 60.6% for HE4, and 94.7% and 76.1% for ROMA, respectively. Both CA125 and HE4 concentrations were significantly higher in patients with primary OC than in women with benign lesions [25]. In addition, Dochez V. et al. [26], who studied the usefulness of CA125, HE4, ROMA, and RMI tests in the diagnosis of ovarian cancer, showed that the most effective diagnostic method to date is the simultaneous determination of CA125 and HE4 [26]. Braicu EI et al. [27] discovered that CA125, HE4, and ROMA exhibit markedly improved diagnostic performance in advanced ovarian cancer (AUC > 0.92) compared to early stage. The researchers found that in the differentiation between OC and endometriosis, both the ROMA (SN 99%) and HE4 (SN 98.1%) performed superiorly compared to CA125 (SN 75.0%) with a specificity rate of 75.4% [27]. A meta-analysis by Li F. et al. [28] showed the predominance of the ROMA test over CA125 and HE4 in predicting EOC (AUC 0.93, 0.88, and 0.82, respectively) [28]. Similar findings were reported by Wang et al. [29] when studying the efficacy of ROMA, CA125, and HE4 in the diagnosis of OC (AUC for ROMA 0.91, for CA125 0.87, and for HE4 0.89). According to the authors, the ROMA algorithm had a higher sensitivity than HE4 and CA125, while HE4 was the most specific for differential diagnosis [29]. Kim B et al. [19] studied a group of Korean women. Comparing the AUCs, they obtained for CA125 AUC 0.811, for HE4 AUC 0.896, for CA125 + HE4 AUC 0.909, for CA125 + HE4 + age AUC 0.892, CA125 + HE4 + menopausal status AUC 0.931, and for CA125 + HE4 + age + menopausal status AUC 0.923. The authors concluded that a complementary approach using CA125, HE4, and ROMA tests is needed to improve the diagnosis of OC [19]. Montagnana et al. [30] reported that ROMA can effectively detect EOC in postmenopausal women, though it does not demonstrate superior efficacy compared to a single HE4 assay [30]. Figure 3 and Figure 4 show a comparison of AUC values for CA125, HE4, and ROMA.

However, when analyzing the usefulness of CA125, HE4, or the ROMA algorithm, the menopausal status of the women in the study group should be taken into account. Analysis of the AUC values for CA125, HE4, and ROMA in the total group shows that the AUC values for the ROMA algorithm are the highest, indicating that a combination of test parameters is better than individual markers.

Zhu C et al. [31] constructed a new diagnostic index, ROMI (Risk of Ovarian Malignancy Index), by combining HE4, CA125, and thymidine kinase 1 (TK1) [31], an enzyme involved in thymidine regeneration for DNA synthesis and DNA damage [32]. Compared to the ROMA algorithm, the ROMI algorithm showed a better diagnostic performance in the differentiation of malignant ovarian tumors from benign lesions. A study showed that TK1 is a potential biomarker for OC. Compared to benign tumors (*p* < 0.001) and controls (*p* < 0.001), TK1 levels were increased in patients with malignant ovarian tumors. The expression of TK1 was positively correlated with the stage of the disease, pelvic metastasis, lymph node metastasis, and distant metastasis (all *p* < 0.001). In premenopausal as well as postmenopausal women, the ROMI index showed higher sensitivity, specificity, and accuracy. The AUC for ROMI also showed higher values than the AUC for ROMA [31].

Another indicator to help diagnose ovarian cancer is the Risk of Malignancy Index (RMI) proposed by Jacobs et al. [33], which considers the sonomorphologic characteristics of the tumor, the age of the patient, and serum CA-125 antigen level. With a RMI threshold of 200, the algorithm had a sensitivity of 85% and specificity of 97% [33], and this value was shown to be the best for distinguishing between benign and malignant lesions, with a SN 51–90% and SP 51–97% [34]. In a paper by Al-Asadi et al. [35], the sensitivity of RMI2 at a cut-off of 200 was 100% and the specificity was 96.2%. According to the researchers, RMI was better at identifying malignant cases than any other single factor in the diagnosis of OC [35]. The usefulness of CA125, HE4, ROMA, RMI, and subjective assessment (SA) in the preoperative diagnosis of ovarian tumors was compared by Janas Ł et al. [36], who obtained AUCs for CA125, HE4, ROMA, RMI, and SA of 0.819, 0.909, 0.911, 0.895, and 0.895, respectively. According to the authors, ultrasound methods are very useful in the diagnosis of ovarian tumors, RMI and SA have a similar overall diagnostic value, while, in their opinion, HE4 and ROMA were the most useful [36]. Van Gorp et al. [37] found that RMI was more helpful than the ROMA algorithm in the diagnosis of ovarian tumors. The researchers also found that ultrasound, especially subjective ultrasound assessment (AUC 0.968) was better than RMI (AUC 0.931) or ROMA (AUC 0.893) in differentiating benign and malignant ovarian tumors [37]. According to Lycke M et al. [38], HE4 is complementary to CA125, especially in identifying benign cases. The investigators concluded that both ROMA and RMI are helpful tools for referring women to a gynecologic oncologist [38]. 

Another multivariate test used in the diagnosis of OC to evaluate the risk index for malignancy is the OVA1 assay, which is composed of five cancer-related proteins: CA125, ApoA-1 (apolipoprotein A-1), TTR (transthyretin), TF (transferrin), and β2-microglobulin. The FDA-approved OVA1 algorithm gave a 96% SN; 28% SP in postmenopausal women and 85% SN; 40% SP in premenopausal women; and detected 76% of malignancies missed by CA125. The FDA also approved the Overa algorithm, which is a combination of CA125-II, HE4, ApoA-1, TF, and folliculotropic hormone. This algorithm showed a SN of 91% and a SP of 69% [39]. A comparison of the above algorithms is shown in Table 1.

Upon analyzing reports on algorithms used for ovarian cancer diagnosis, it is noted that the RMI and ROMI algorithms have the highest sensitivity and specificity values. The researchers suggested that RMI is the most effective single factor for identifying malignant cases in the diagnosis of OC, and that it is more helpful than the ROMA algorithm in diagnosing ovarian tumors.

## 4. CA125 and Its Use in Combination with Other Markers 

To improve the effectiveness of CA125 as a screening test for ovarian cancer, models combining this marker with other biomarkers and transvaginal ultrasound are being investigated. It is difficult to find a strategy with a high level of sensitivity and specificity. To achieve the highest sensitivity and specificity, individual markers are combined into diagnostic panels. Attempts are being made to create combinations of CA125 with HE4, CA72-4, mesothelin, transthyretin, or osteopontin, among others. A comparison of the sensitivity and specificity of selected individual parameters is shown in Table 2.

Given that an ideal screening test should have a sensitivity of greater than 75% and a specificity of at least 99.6% [5,6], it is concluded that none of the individual tests listed meet these requirements. Kozak KR et al. [44] concluded that combining CA125, TTR, Hb, ApoAI, and TF is expected to significantly improve early ovarian cancer detection. According to Matsas et al. [45] the use of a combination of CA-125, CA15-3, CA 19-9, HE4, and hCG (human chorionic gonadotropin) has the potential to improve early detection of the disease and can make a valuable contribution to diagnosing, monitoring, and prognosticating ovarian cancer [45]. Yurkovetsky Z et al. [46] created a set of biomarkers consisting of CA-125, HE4, CEA, and VCAM-1 (VCAM-1-vascular cell adhesion molecule-1). This combination provided the best diagnostic power, with an SN of 86% for early stage OC and an SN of 93% for late stage OC with an SP of 98% [46]. Whitwell HJ et al. [47] studied serum samples from women who were later diagnosed with ovarian cancer and serum samples from control women who did not have cancer. The researchers tested combinations of multimarkers, the best of which included CA125, HE4, CHI3L1 (chitinase-3-like protein 1), PEBP4 (phosphatidylethanolamine-binding protein 4), and/or AGR2 (anterior gradient protein 2), providing 85.7% SN with 95.4% SP up to one year before diagnosis. For type II cancer (mainly high-grade serous carcinoma), the combinations achieved a SN of 95.5%, the SP was 95.4%, and predictive values were increased earlier than CA125. The combinations significantly outperformed CA125 in detecting type I and type II cases that CA125 failed to detect. The researchers emphasized, however, that these studies still need independent validation [47]. In ovarian cancer samples with serous and endometrioid histology, Nosov V et al. [48] studied a set of four serum parameters: ApoA-1, TTR, TF, and CA-125. This panel detected early stages of OC with 96% SN and 96% SP. The authors stressed that the panel needs to be analyzed in a prospective clinical setting [48]. Rani S et al. [49] conducted a study on 106 women, of whom 26 had ovarian cancer, 31 had benign ovarian tumors, and the control group consisted of 49 women. The purpose of the research was the evaluation of CA125 and osteopontin (OPN) for the detection of malignant tumors. The researchers found that the CA125 marker was more sensitive than OPN, but the specificity of OPN was much better, so this parameter could better distinguish between benign and malignant ovarian tumors. The sensitivity of CA125 improved when combined with OPN, but the specificity of the combination decreased significantly [49]. O’Shannessy et al. [50] evaluated and compared the usefulness of serum FRA (folate receptor alpha) and MPF (megakaryocyte enhancer factor) assays with CA125, MSLN (mesothelin), and HE4 assays in the diagnosis of EOC. According to the investigators, the best combination was CA125, FRA, MSLN, and MPF, whose ability to differentiate between ovarian cancer patients and healthy women was significantly better than CA125 alone (AUC = 0.91, *p* < 0.0001 vs. CA125 AUC = 0.84, *p* < 0.0001) [50]. The utility of a CM (composite marker), a combination of CA125 and SMR (soluble mesothelin), was evaluated by McIntosh et al. [51]. The researchers showed that the CM had the best sensitivity, with a specificity equal to that of CA125. The study showed that the composite marker identified 86.5% of cases (45 of 52) in comparison to the 78.8% (41 of 52) achieved by CA125, with a specificity of 98% [51]. Table 3 compares the sensitivity and specificity of CA125 alone and in combination with selected markers. 

CA125 is a commonly used marker in the diagnosis of ovarian cancer. However, it lacks the specificity and sensitivity required for reliable early detection of the disease, as only 50% of early-stage ovarian cancer cases have elevated CA125 levels. Additionally, elevated CA125 levels can be associated with many other conditions [18]. Other markers, such as HE4, or algorithms, such as ROMA, are also used for diagnosis. However, the search for a marker or combination of markers that meets the criteria of an ideal marker is ongoing. When comparing the sensitivity/specificity of a single CA125 test and the sensitivity/specificity of combinations of CA125 with other markers, we see that in most cases combinations perform better than a single test. It is very difficult to select parameters that meet the criteria of an ideal screening test (SN 75%, SP 99.6%). Among the combinations analyzed in this article, two sets meet these requirements: CA125 + HE4 + sEGFR (SN 83.3%, SP 100%) and CA-125 + HE4 + E-CAD + IL-6 (SN 86.4%, SP 100%).

## 5. CA125 and Potential Biomarkers

Modern research techniques based, among other things, on molecular biology allow a continuous expansion of research aimed at finding more and more effective biomarkers for ovarian cancer. There is a search for a complementary parameter to the most commonly used CA125 or HE4, which would satisfactorily increase the sensitivity and specificity of the determinations. Among potential biomarkers, microRNAs, autoantibodies, cDNAs, adipocytokines, and galectins are drawing the attention of researchers. 

### 5.1. MicroRNAs 

MicroRNAs are small, non-coding, single-stranded RNA molecules (21–23 nucleotides). They play a crucial role in regulating gene expression, and their altered expression has been linked to various diseases [57,58]. The expression of some miRNAs is inhibited in ovarian cancer, suggesting that they can be considered suppressor genes, while other miRNAs undergo abnormal expression and can be considered ovarian cancer-promoting genes [59]. These molecules are readily available markers, because the miRNA profile in plasma often mirrors miRNAs in tumor tissue [60]. According to Zhao L. et al. [61], miRNA molecules are potential biological targets for early detection, targeted therapy, drug-resistance monitoring, and prognosis improvement in ovarian cancer [61]. Meng X et al. [62] showed that a set of molecules: miR-200a, miR-200b, and miR-200c had a SN of 83% and SP of 100% in differentiating malignant and benign ovarian tumors [62]. Ali F.T. et al. [2] compared miRNA-204 with CA125 and CA 19-9. The sensitivity of the biomarkers used was 98.04%, 100.00%, and 96.19%, respectively, and the specificity was 58.33%, 62.50%, and 57.78%, respectively. The AUCs for each marker in patients with early ovarian cancer were miRNA-204 AUC = 0.924, for CA125 AUC = 0.926, and for CA19-9 AUC = 0.914, while for the combination of these parameters AUC = 1.000. The researchers concluded that combining these three biomarkers is a good diagnostic tool in ovarian cancer. [2]. On the other hand, Zhu Z. et al. [63] tested a set consisting of CA125 with HE4 and exosomal miR-205. The SN of the assay was 100% and the specificity was 86.1% [63]. Other investigators combined the CA-125 assay with six miRNA molecules: miR-200a-3p, miR-766- 3p, miR-26a-5p, miR-142-3p, let-7d-5p, and miR-328-3p. The combination showed an AUC of 0.994 (95% CI, 0.988–0.999), a sensitivity of 0.984 and a specificity of 0.956, at the optimal cut-off points [64]. Su et al. [65] described that a set of miR-1307 and miR-375 molecules with CA-125 and HE4 significantly increased the diagnostic accuracy of ovarian cancer. The AUC for the miR-1307 + CA125 + HE4 combination was 0.970 and the AUC for the miR-375 + CA125 + HE4 combination was 0.945 [65]. Similarly, Oliveira et al. [66] achieved significant diagnostic accuracy by combining miR-200c-3p and miR-221-3p molecular expression assays with the conventional CA-125 test: AUC = 0.96 in OC. Their results suggest that the combination of miR-200c-3p, -221-3p and CA-125 may improve the differentiation between late-stage OC and mild cases of OC [66]. The diagnostic utility of selected combinations of CA125 and miRNAs are compared in Table 4.

### 5.2. Autoantibodies (AAbs)

The use of proteomics allows the identification of new protein biomarkers, the search for proteins related to human immunoglobulins, and the detection of autoantibodies (AAbs) [40]. AAbs can be present in higher concentrations than the equivalent antigen, show better hold up over time, and may be able to be detected at an earlier stage of the disease. The limited data available suggest that selected AAb markers may improve diagnostic value when used in combination with CA125 and HE4 [67]. Lu D et al. [68] investigated 14 biomarkers associated with ovarian cancer, including CA125, MIF- (1macrophage inhibitory factor-1), leptin, prolactin, osteopontin, IGF-II (insulin-like growth factor-II), and autoantibodies to eight proteins: p53, NY-ESO-1, p16, ALPP, CTSD, B23, GRP78, and SSX. The trial was carried out in 151 patients with OC, 23 with borderline tumors, 55 with benign tumors, and 75 healthy controls. According to the researchers, p53 AAbs in combination with CA125 could be a useful biomarker for the detection of ovarian cancer type II [68]. Yang WL et al. [69] evaluated the benefit of combining the CA125 marker with the TP53 autoantibody for the detection of invasive epithelial ovarian cancer. The authors found that the AUC was significantly higher for the TP53 and CA125 autoantibody combination (AUC for TP53 was 0.699, AUC for CA125 was 0.838, while AUC for TP53 AAb + CA125 was 0.867, *p* = 0.007) [69]. Lokshin AE et al. [70] found that serum IL-8 and anti-IL-8 antibody levels were elevated in ovarian cancer patients compared to healthy controls, and that combining IL-8 and anti-IL-8 IgG with CA125 resulted in increased classification power compared to each marker analyzed separately. The researchers conclude that IL-8 and anti-IL-8 autoantibodies may be used as additional biomarkers for OC [70].

Duan Y et al. [71] evaluated serum autoantibodies against tumor-related such as LRDD (leucine repeat death domain containing protein), STC1 (Stanniocalcin-1) and FOXA1 (Forkhead-box A1). Their study showed that these autoantibodies have a high diagnostic value and can complement other serum biomarkers used to detect OC. The authors concluded that the combined use of anti-LRDD and anti-FOXA1 autoantibodies and CA125 is promising for the detection of OC in clinical practice [71]. A study by Qiu C et al. [72] suggested that autoantibodies to PDLIM1 may have potential as a new serologic biomarker for OC, complementary to CA125 assays, which may improve the power of OC detection [72]. A comparison of the diagnostic utility of selected combinations of CA125 and autoantibodies is shown in Table 5.

### 5.3. ctDNA

Other molecules being considered as potential adjuncts to CA125 determinations include ctDNA, fragments of DNA that are released from tumor tissues into blood, urine, and other body fluids by apoptosis, necrosis, lysis, and active secretion [74]. Li B et al. [75], in a meta-analysis based on 22 previous studies, showed that circulating ctDNA has comparable efficacy to CA125 and HE4 (AUCs of 0.8958, 0.883, and 0.899, respectively) [75].

### 5.4. Adipocytokines

Adipocytokines are hormonally active proteins with autocrine, paracrine, and endocrine effects. They are secreted by adipose tissue [76]. Hasenburg A et al. [77] researched the usefulness of a panel consisting of CA125, HE4, OPN, leptin, and prolactin in the differentiation between benign and malignant ovarian tumors. The researchers found that that combining the above five proteins outperformed the single marker CA125 in its diagnostic ability in ovarian cancer. The AUC of the panel was 0.96 compared to the AUC for CA125 of 0.929 [77]. Other investigators developed a multiplex assay consisting of six serum biomarkers including leptin, prolactin, osteopontin, IGF-II (insulin-like growth factor II), MFI (macrophage inhibitory factor), and CA-125. Combining these proteins resulted in 95.3% sensitivity and 99.4% specificity [78]. Matte et al. [79] studied patients diagnosed with HGSOC (high-grade serous ovarian cancer) with baseline clinical resistance to platinum-based therapy. The investigators correlated serum and ascites CA125 marker levels with ascites leptin levels. They showed that high serum CA125/ascites leptin ratios were associated a shorter PFS and a poorer OS. According to the authors, this ratio may be a predictive biomarker for OC, but this requires further analysis [79]. Li H et al. [80] analyzed the correlation of ADPN (adiponectin), plasma D-Dimers, inflammatory factors, CA125, HE4, and ROMA. The authors found that, in ovarian cancer, ADPN levels decrease, while D-D levels, inflammatory factors, and tumor markers increase significantly. In addition, ROMA shows a positive association with the levels of CA125, HE4, plasma D-D, and inflammatory factors, and a negative association with ADPN levels [80]. An inverse correlation between CA125 and ITLN1 (omentin) levels was detected by Au-Yeung et al. [81]. The authors found that CA125 had a significantly larger AUC than omentin (*p* = 0.0031). However, the combined parameters had significantly larger AUCs than CA125 alone (*p* = 0.0295) or ITLN1 alone (*p* = 5.095 × 10^−6^). According to the authors, ITLN1 complements CA125 in the identification of OC patients [81]. 

The diagnostic utility of CA125 and selected adipocytokines are compared in Table 6.

### 5.5. Galectins (Gal)

A group of proteins called galectins (Gal) is also of interest to researchers. Selected galectins have been observed to be involved in the development and progression of ovarian cancer. From previous studies, galectins appear to be a useful diagnostic and prognostic tool for assessing tumor progression and the efficacy of therapy in patients with ovarian cancer. However, more research is needed [82,83]. The usefulness of galectin-1 as a biomarker for ovarian cancer was investigated by Masoodi M et al. [84]. The analysis of the ROC curve (receiver operating characteristic curve) showed an AUC of 0.936 for galectin-1 and an AUC of 0.89 for CA-125. With a cut-off value of >15.9 ng/mL, galectin-1 had a SN of 88.89% and a SP of 93%, while with a cut-off value of >36.5 ng/mL for CA-125, a SN of 80.65% and a SP of 89% were reported. The researchers emphasized the clinical importance of serum galectin-1 as a non-invasive biomarker for the early detection of OC, for the monitoring of response to treatment, and as an indicator of tumor metastasis and invasion [84]. The predictive potential of galectin in relation to the commonly used CA125 was investigated by Labrie M et al. [85]. High CA125 levels (≥412 U/mL) were a risk factor for both 5-year DFS (disease-free survival) and 5-year OS. Galectins did not alter prognosis in patients with high plasma CA-125 levels. However, stromal gal-1 (*p* = 0.041) and epithelial gal-9 (*p* = 0.005) had significant predictive value in patients with low plasma CA-125 levels. According to the authors, the expression of gal-1 in the stromal cells and of gal-8 and gal-9 in the epithelial tumor cells has a strong prognostic potential as a molecular marker for HGSC [85]. Abdelwahabi et al. [86] measured CA125 and Gal-1 levels in women with different stages of SOC (serous ovarian cancer). They found that serum Gal-1 at a cut-off >135 ng/mL was superior to CA-125 at a cut-off >49 u/mL with SN 100% and SP 100%, vs. SN 88.57% and SP 96% for CA-125. Serum Gal-1 concentration was significantly associated with tumor stage (*p* < 0.001) [86].

Of the potential biomarkers, we consider miRNA molecules to be the most noteworthy. The combination of miR-200a, miR-200b, and miR-200c achieved a sensitivity of 83% and a specificity of 100%. Antibodies are also promising, with the combination of CA125 + p53 AAb giving a sensitivity of 85.7% and a specificity of 100%. Both galectin family proteins and adipocytokines, as well as cDNA molecules, show promise as markers for diagnosing ovarian cancer. Their inclusion in diagnostic assays significantly improves sensitivity and specificity compared to using the single CA125 assay.

## 6. Conclusions

It is difficult to compare sensitivity, specificity, or AUC between the different tests described because of differences in the types of benign gynecological conditions used as controls, the stages and pathological types of ovarian cancer patients, the menopausal status of patients, or the cut-off point for diagnosing ovarian cancer. The researchers tried to find a parameter that could be added to the most commonly used parameters that would satisfactorily increase the sensitivity and specificity of tests useful in the early stages of ovarian cancer. This review examined combinations of parameters used to diagnose ovarian cancer and compared the usefulness of the tests by comparing sensitivity, specificity, or AUC. Combinations of parameters were found to perform better than single tests. However, further trials with a large number of patients would be required to confirm the usefulness of any particular set of tests. Improving the detection of ovarian cancer and thereby reducing mortality among women has become a priority in modern gynecologic oncology. Numerous studies are underway, with the overarching goal of finding new markers and combinations of markers that if used at an early stage of the disease would be an effective screening test and help develop novel diagnostic and therapeutic strategies for ovarian cancer patients.

## Figures and Tables

**Table 1:** Comparison of sensitivity and specificity of algorithms used in the diagnosis of ovarian cancer [6,31,33,35,36,39,40].

Parameter	SN	SP	Ref
RMI I	78%	87%	[6]
RMI II	79%	81%	
RMI III	74%	91%	
RMI	71%	97%	[40]
	88%	74%	
RMI	85%	97%	[33]
RMI2	100%	96.2%	[35]
ROMA			[36]
premenopausal	76.47%	84.13%	
postmsenopausal	79.05%	77.78%	
ROMI			[31]
premenopausal	84.6%	93.9%	
postmsenopausal	98.2%	97.0%	
OVA1			[39,40]
premenopausal	85%	40%	
postmenopausal	96%	28%	
OVERA	91%	69%	[39]

**Table 2:** Comparison of sensitivity and specificity of selected individual parameters in the diagnosis of ovarian cancer [38,41,42,43].

Parameter	SN	SP	Comments	Ref
CA125	92%	80%	for early detection of ovarian cancer	[38]
	7.7%	98%	for early detection of ovarian cancer	[41]
	23.9%	98%	for all types and stages of ovarian cancers	[41]
	73%	95%	all cases and general population controls	[43]
	56%	95%	early stage cases and general population controls	[43]
HE4	71.19%	85%	for early detection of ovarian cancer	[42]
	30.8%	98%	for early detection of ovarian cancer	[41]
	64.2%	98%	for all types and stages of ovarian cancers	[41]
transthyretin	47%	95%	all cases and general population controls	[43]
	29%	95%	early stage cases and general population controls	
osteopontin	7.6%	98%	for early detection of ovarian cancer	[41]
	4.9%	98%	for all types and stages of ovarian cancers	
soluble mesothelin-related peptide (MSRP)	15.4%	98%	for early detection of ovarian cancer	[41]

**Table 3:** Sensitivity and specificity of selected parameter combinations useful in the diagnosis of ovarian cancer. A comparison was made between the sensitivity and specificity of a single CA125 assay and combinations of CA125 and selected markers [14,44,46,49,52,53,54,55,56].

Parameter	Sensitivity SN	Specitivity SP	Ref
CA125	93.33%	85.11%	[14]
CA125 + HE4	86.67%	95.75%	
CA125 + HE4 + sEGFR (in the diagnosis of EOC)	83.30%	100.00%	
CA125	79.5%	85%	[52]
CA125 + HE4	83%	85%	
CA125 + HE4 + sEGFR	83.3%	85%	
CA-125 + HE4 + CEA + Cyfra 21-1	86.4%	85%	
CA-125	61%	98%	[46]
CA-125 + HE4 + CEA + VCAM-1 (for early stage)	86%	98%	
CA125	74.2%	94.9%	[53]
CA125 + CYFRA21-1 (for EOC)	82.8%	55.7%	
CA125	67%	95%	[54]
CA125 + TTR + ApoA1	95.5%	95%	
CA125	30%	95%	
CA125 + TTR + ApoA1 (for stage I + II)	93.9%	95%	
CA125	91.6%	95%	
CA125 + TTR + ApoA1 (for stage III + IV)	96.5%	95%	
CA125	78%	78%	[44]
CA125 + TTR+ Hb + ApoAI + TF (for detection of early stage ovarian tumors in all histological groups)	86%	86%	
CA125	41.2%	83.4%	[55]
CA125 + CA15-3 + CA72-4 (for total group)	88.6%	71.6%	
CA125	88.5%	61.3%	[49]
CA-125 + OPN	93%	39.3%	
CA125	90.4%	87%	[56]
CA-125 + HE4 + E-CAD + IL-6	86.4%	100%	

sEGFR—Soluble epidermal growth factor receptor, CEA—carcinoembryonic antygen, Cyfra21-1—Cytokeratin 19 fragment, VCAM-1—vascular cell adhesion molecule-1, TTR—Transthyretin, ApoA1—apolopoprotein A-I, TF—transferrin, CA15-3—cancer antigen 15-3, CA72-4—cancer antigen 72-4, OPN—osteopontin, IL-6—interleukin 6.

**Table 4:** Comparison of the diagnostic utility of selected combinations of CA125 and miRNAs [2,62,63,64,65,66].

Parameter	SN	SP	AUC	Ref
CA125	85	85		[66]
miR-221 + miR200c	52	84		
CA125 + miR-221 + miR200	91	84	0.96	
miR-200a, miR-200b and miR-200c	83	100		[62]
miRNA-204	98.04	58.33	0.942	[2]
CA125	100.00	62.50	0.980	
CA 19-9	96.19	57.78	0.918	
miRNA-204 + CA125 + CA 19-9			0.998	
CA125	74.3	93.7	0.915	[63]
CA125 + HE4 + exosomal miR-205	100	86.1	0.951	
Mir-1307 + CA125 + HE4	-	-	0.970	[65]
Mir-375 + CA125 + HE4	-	-	0.945	
CA-125 + miR-200a-3p, miR-766-3p, miR-26a-5p, miR-142-3p, let-7d-5p and miR-328-3p.	98.4	95.6	0.994	[64]

CA 19-9—cancer antigen 19-9.

**Table 5:** Comparison of the diagnostic utility of selected combinations of CA125 and autoantibodies [68,69,70,73].

Parameter	SN	SP	AUC	Ref
CA125			0.838	[69]
CA125 + TP53AAb			0.867	
CA125	73.8	100	0.9310	[68]
CA125 + p53 AAb	85.7	100	0.9694	
CA125	76.8	98		[70]
IL-8	62.6	98		
anty-IL 8	65.6	98		
anty-IL-8 + IL-8 + CA125	87.5	98		
CA125	76	95	0.93	[73]
CA125 + BARD1	91	95	0.98	

**Table 6:** Comparison of the diagnostic utility of CA125 and selected adipocytokines [77,78,81].

Parameter	SN	SP	AUC	Ref
CA125			0.929	[77]
CA125, HE4, OPN, leptin, prolactin			0.96	
leptin, prolactin, OPN, IGF-II, MIF, CA-125	95.3%	99.4%		[78]
CA125			0.9287	[81]
CA125 + ITLN1			0.9664	

IGF-II-insulin-like growth factor II, MIF—macrophage inhibitory factor.

## Data Availability

Not applicable.
